# Cationic Noble-Gas Hydrides: From Ion Sources to Outer Space

**DOI:** 10.3389/fchem.2020.00462

**Published:** 2020-06-19

**Authors:** Felice Grandinetti

**Affiliations:** ^1^Dipartimento per la Innovazione nei Sistemi Biologici, Agroalimentari e Forestali (DIBAF), Università della Tuscia, Viterbo, Italy; ^2^Istituto per i Sistemi Biologici del CNR, Monterotondo, Italy

**Keywords:** noble-gas ions, noble-gas chemistry, interstellar chemistry, gas-phase chemistry, mass spectrometry, theoretical calculations

## Abstract

Cationic species with noble gas (Ng)-hydrogen bonds play a major role in the gas-phase ion chemistry of the group 18 elements. These species first emerged more than 90 years ago, when the simplest HeH^+^ and HeH${}_{2}^{+}$ were detected from ionized He/H_2_ mixtures. Over the years, the family has considerably expanded and currently includes various bonding motifs that are investigated with intense experimental and theoretical interest. Quite recently, the results of these studies acquired new and fascinating implications. The diatomic ArH^+^ and HeH^+^ were, in fact, detected in various galactic and extragalactic regions, and this stimulates intriguing questions concerning the actual role in the outer space of the Ng-H cations observed in the laboratory. The aim of this review is to briefly summarize the most relevant information currently available on the structure, stability, and routes of formation of these fascinating systems.

About 130 years after the discovery of argon (Rayleigh, 1895), the chemistry of the noble gases currently appears as a fascinating “saga” (Grandinetti, 2018), where combative scientists never tire of using different chemical and physical weapons to challenge and defeat the proverbial inertness of the elements. Exemplary in this regard is the unceasing interest focused on gaseous ionic species. The story began in 1925, when Hogness and Lunn (1925) first detected the simplest HeH^+^ and HeH${}_{2}^{+}$ from ionized He/H_2_ mixtures. With the upsurge of interest in gas-phase ion-molecule reactions (Stevenson, 1957; Giese et al., 1961), it soon emerged that, under ionizing conditions, all the noble gas atoms (Ng) were “forgetting” to be inert, and capable of combining with a huge variety of atoms and molecules. The subsequent studies actually confirmed an exciting ion chemistry of both fundamental and applied interest (Grandinetti, 2011). None could, however, surmise the amazing implications that emerged quite recently, when ArH^+^ and HeH^+^ were detected in various galactic and extragalactic regions (Barlow et al., 2013; Schilke et al., 2014; Müller et al., 2015; Güsten et al., 2019). This unexpected projection from the ion sources to outer space rejuvenates particular interest in cationic noble-gas hydrides Ng_*m*_H_*n*_^+^ (*m, n* ≥ 1), a well-established family of noble gas ions. The pertinent literature is already vast, and the contributions chosen here wish to illustrate issues of current interest that are also of relevance for the naturally-occurring chemistry. The systems are described so as to give an overview, useful also as a guide for future studies.

## NgH^+^

**Figure 1:**
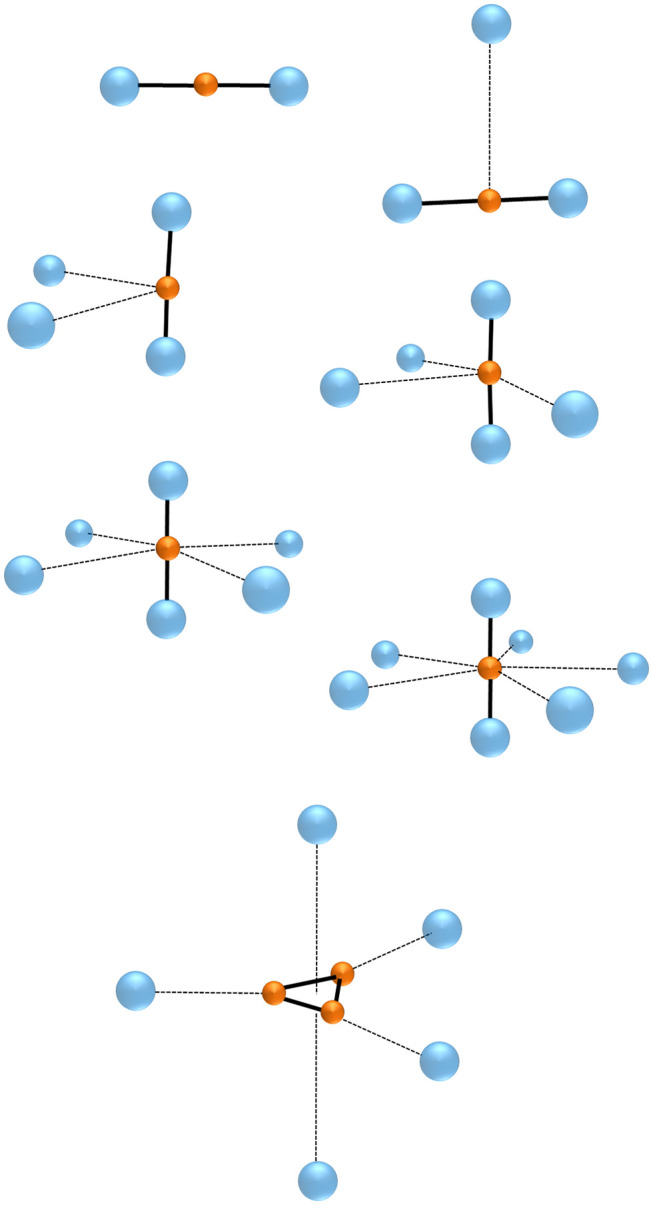
Connectivities of the Ng-H-Ng^+^, (Ng-H-Ng^+^)(Ng)_*n*_ (*n* = 1–5), and H${}_{3}^{+}$(Ng)_5_.

The chemistry of the gaseous Ng_*m*_H_*n*_^+^ plays around four major bonding motifs, namely the NgH^+^, the linear centro-symmetric Ng-H-Ng^+^, and their Ng-solvated complexes (NgHNg^+^)(Ng)_*n*_, the (H${}_{2}^{+}$)(Ng)_*n*_, and the (H${}_{3}^{+}$)(Ng)_*n*_ (*n* ≥ 1). Their formations also benefit from ion sources operated at ultra-cold temperatures (Jašik et al., 2013), or from ionized helium nanodroplets doped with suitable precursors (Fárník and Toennies, 2005). The connectivities of some exemplary species are shown in Figure 1, and some quantitative data are reported in Table 1.

**Table 1:** Energetics (kcal mol^−1^) of reactions involving the gaseous Ng_*m*_H_*n*_^+^, and bond distances (*R*, Å) and vibrational frequencies (ν, cm^−1^) of the NgH^+^ and Ng-H-Ng^+^.

**Reaction^a^**	**He**	**Ne**	**Ar**	**Kr**	**Xe**
Ng + H^+^ → NgH^+^	−42.5	−47.5	−88.2	−101.5	−119.4
Ng^+^ + H → NgH^+^	−295.9	−231.2	−138.0	−110.7	−85.5
Ng + H${}_{2}^{+}$ → NgH^+^ + H	19.6	14.6	−26.1	−39.4	−57.3
Ng^+^ + H_2_ → NgH^+^ + H	−191.7	−127.0	−33.8	−6.5	18.7
Ng + NgH^+^ → Ng-H-Ng^+^	−13.2^b^	−15.6^c^	−14.8^c^	−14.6^c^	−13.9^c^
Ng + H${}_{2}^{+}$ → Ng-H-H^+^	−7.8^d^	−12.9^e^	−42.4^e^		
2Ng + H${}_{2}^{+}$ → Ng-H-H-Ng^+^^f^	−5.5	−13.1	−50.0	−66.4	−89.9
Ng + H${}_{3}^{+}$ → Ng(H${}_{3}^{+}$)	−0.22 ÷−0.41^g^	−2.5^h^/−2.6^i^	−6.7^j^/−8.7^h^/-9.0^i^	−13.9^h^	−22.8^h^
**NgH**^**+**^^k^					
*R*	0.774^l^	0.991^m^	1.280^m^	1.421^m^	1.603^m^
ν	2911^n^	2904^m^	2711^m^	2495^m^	2270^m^
**Ng-H-Ng**^**+**^					
*R*	0.926^o^	1.144^p^	1.502^p^	1.661^p^	1.862^p^
ν${}_{3}^{\text{q}}$	1290 (gas)^r^	1432^s^	1000^s^ 989 (gas)^t^ 903 (Ar)^u^	927^s^ 853 (Kr)^u^ 885 (Ar)^v^ 871 (*p*-H_2_)^w^ 871 (*n*-H_2_)^w^	842^s^ 731 (Xe)^u^ 828 (Ar)^v^ 782 (Kr)^v^ 847 (*p*-H_2_)^w^ 845 (*n*-H_2_)^w^
ν${}_{1}^{\text{x}}$ + ν_3_		1814^s^	1253^s^ 1237 (gas)^t^ 1140 (Ar)^u^	1092^s^ 1008 (Kr)^u^ 974 (*p*-H_2_)^w^ 985 (*n*-H_2_)^w^	964^s^ 843 (Xe)^u^ 953 (Ar)^v^ 900 (Kr)^v^ 972 (*p*-H_2_)^w^ 965 (*n*-H_2_)^w^
2ν_1_ + ν_3_		2182^s^	1500^s^ 1485 (gas)^t^ 1361 (Ar)^u^	1257^s^ 1160 (Kr)^u^	1085^s^ 953 (Xe)^u^
3ν_1_ + ν_3_		2541^s^	1750^s^ 1726 (gas)^t^	1428^s^ 1309 (Kr)^u^	1213^s^ 1062 (Xe)^u^
4ν_1_ + ν_3_			2401 (gas)^t^		1168 (Xe)^u^

a *Unless stated otherwise, experimental enthalpy changes (ΔH) from National Institute of Standards and Technology (2020)*.

b *CCSD(T)/CBS electronic energy change (ΔE_el_) from Kim and Lee (1999)*.

c *CCSD(T)/aug-cc-pVQZ//MP2/aug-cc-pVQZ energy change at 0 K [ΔE(0)] from Tan and Kuo (2019)*.

d *FCI/aug-cc-pV5Z ΔE_el_ from Koner et al. (2019)*.

e *CCSD(T)/aug-cc-pVTZ ΔE_el_ from Theis et al. (2015)*.

f *CCSD(T)/def2-TZVPP ΔE(0) from Krapp et al. (2008)*.

g *Range of theoretical values quoted by Savić et al. (2015)*.

h *CCSD(T)/aug-cc-pVQZ ΔE_el_ from Pauzat et al. (2016)*.

i *CCSD(T)/aug-cc-pV5Z ΔE_el_ from Theis and Fortenberry (2015)*.

j *ΔH from Hiraoka and Mori (1989)*.

k *Gas-phase*.

l *Coxon and Hajigeorgiou (1999)*.

m *Rogers et al. (1987)*.

n *Perry et al. (2014)*.

o *CCSD(T)/aug-cc-pVTZ value from Kim and Lee (1999)*.

p *MP2/aug-cc-pVQZ value from Tan and Kuo (2019)*.

q *Anti-symmetric stretching*.

r *Gas-phase value from Asvany et al. (2019)*.

s *Discrete variable representation (DVR) theoretical value based on a CCSD(T)/aug-cc-pVQZ//MP2/aug-cc-pVQZ potential from Tan and Kuo (2019)*.

t *Gas-phase value from McDonald et al. (2016)*.

u *Value in Ar, Kr, or Xe matrix from Kunttu and Seetula (1994)*.

v *Value in Ar or Kr matrix from Lundell et al. (1999)*.

w *Value in H_2_ matrix from Tsuge et al. (2015)*.

x *Symmetric stretching*.

The diatomic NgH^+^ (Ng = He-Xe), still elusive in any other environment, are quite stable in the gas phase. Their simplest ionic routes of formation given by the equations

(1) $$\begin{array}{r} \text{Ng}+{\text{H}}^{+}\to NgH^{+} \end{array}$$

(2) $$\begin{array}{r} \text{N}{\text{g}}^{+}+H\to NgH^{+} \end{array}$$

(3) $$\begin{array}{r} \text{Ng}+{\text{H}}_{2}^{+}\to NgH^{+}+H \end{array}$$

(4) $$\begin{array}{r} \text{N}{\text{g}}^{+}+H_{2}\to NgH^{+}+H \end{array}$$

are, invariably, exothermic (National Institute of Standards and Technology, 2020), with the only exception of He and Ne reacting with ground-state H${}_{2}^{+}$ (see Table 1). The galactic and extragalactic ArH^+^ is, in particular, ascribed to reactions (3) and (4) (Barlow et al., 2013; Theis et al., 2015), whereas the HeH^+^ observed in the planetary nebulae (Güsten et al., 2019) mainly arises from reaction (2) (Roberge and Dalgarno, 1982; Cecchi-Pestellini and Dalgarno, 1993; Fortenberry, 2019). In keeping with any type of bonding analysis (Borocci et al., 2015), the short bond distances, and high vibrational frequencies of the NgH^+^ point to typical covalent species. The experimental values (Rogers et al., 1987, and references cited therein; Coxon and Hajigeorgiou, 1999; Perry et al., 2014; Gruet and Pirali, 2019) (see Table 1) range between 0.774 and 1.603 Å, and 2,911 and 2,270 cm^−1^, respectively, and follow the expected periodic increase/decrease of *R*/ν when going from HeH^+^ to XeH^+^.

## Ng-H-Ng^+^ and (NgHNg^+^)(Ng)*_*n*_* (*n* ≥ 1)

The addition of another Ng to NgH^+^ produces the linear centro-symmetric Ng-H-Ng^+^. Their thermochemistry is experimentally unknown, but theoretical calculations (Kim and Lee, 1999; Tan and Kuo, 2019) predict Ng additions that are exothermic by ca. 13–17 kcal mol^−1^. The alternative conceivable Ng-Ng-H^+^ are instead only marginally stable (Bop et al., 2017a,b), and do not play a role in the chemistry and the dynamics of the gaseous H^+^(Ng_*m*_) (Ritschel et al., 2004, 2005, 2007). The structural assignment of the Ng-H-Ng^+^ is based first on matrix infrared (IR) spectroscopy. Definitive evidence in this regard was obtained, in particular, by Kunttu and his coworkers (Kunttu et al., 1992; Kunttu and Seetula, 1994), who identified the symmetric (ν_1_) and anti-symmetric (ν_3_) stretching that appear in the IR spectra of the Ng-H-Ng^+^ (Ng = Ar, Kr, Xe) as a progression of (*n*ν_1_ + ν_3_), with *n* up to 4 for Xe-H-Xe^+^. The ν_1_ and (ν_1_ + ν_3_) absorptions of Kr-H-Kr^+^ and Xe-H-Xe^+^ were subsequently measured in different matrices (Lundell et al., 1999; Tsuge et al., 2015), and found to be sensitive to the environment. As shown in Table 1, the comparison with very recent theoretical estimates (Tan and Kuo, 2019) clearly unravels that the “cold” bands are red-shifted by up to 100 cm^−1^ with respect to those of the naked Ng-H-Ng^+^. Consistently, the theoretical IR absorptions of Ar-H-Ar^+^ are instead quite close to the corresponding gas-phase values obtained by Duncan and his coworkers (McDonald et al., 2016). They produced the entire family of the smallest (Ar-H-Ar^+^)Ar_*n*_ (*n* = 1–5), and measured the IR photodissociation spectrum (loss of one Ar atom) of each mass-selected complex. In general, the (Ng-H-Ng^+^)Ng_*n*_ consist (Giju et al., 2002; Ritschel et al., 2005, 2007; Császár et al., 2019) of Ng atoms weakly bound to a covalent centro-symmetric Ng_2_H ^+^, the (Ng-H-Ng^+^)Ng_5_ growing by the step-by-step addition of five Ng in the plane perpendicular to the Ng-H-Ng^+^ axis (see Figure 1). The IR patterns of the gaseous (Ar-H-Ar^+^)Ar_*n*_ were assigned (McDonald et al., 2016) as the ν_3_ and (*n*ν_1_ + ν_3_) progression, with *n* arriving up to 4 for (Ar-H-Ar^+^)Ar. As shown in Table 1, the ν_3_ (989 cm^−1^), (ν_1_ + ν_3_) (1,237 cm^−1^), and (2ν_1_ + ν_3_) (1,485 cm^−1^) absorptions of the latter species are, invariably, blue-shifted with respect to the corresponding values measured in argon matrix. Consistently, when going from (Ar-H-Ar^+^)Ar to (Ar-H-Ar^+^)Ar_5_, the ν_3_ and (ν_1_ + ν_3_) resulted progressively red-shifted up to 965 and 1,207 cm^−1^, respectively. A strictly similar trend was quite recently noticed by Asvany et al. (2019), who measured the ν_3_ of the gaseous (He-H-He^+^)He_*n*_ (*n* = 1–4) ranging between 1,290 (*n* = 1) and 1,273 cm^−1^ (*n* = 4). As for the larger (Ng-H-Ng^+^)Ng_*n*_ (*n* > 5), the available experimental results (Kojima et al., 1992; Bartl et al., 2013; Gatchell et al., 2018, 2019; Lundberg et al., 2020) unravel intriguing differences between the heaviest (Ng-H-Ng^+^)Ng_*n*_ (Ng = Ne, Ar, Kr), and the He congeners. For the former species, the mass-spectrometric abundancies (Gatchell et al., 2018, 2019) indicate the “magic” role of three structures, namely the (Ng-H-Ng^+^)Ng_5_, the (Ng-H-Ng^+^)Ng_11_, and the (Ng-H-Ng^+^)Ng_17_. Based also on theoretical calculations, these species are identified as the most stable intermediates along a icosahedral growth. Starting from the (Ng-H-Ng^+^)Ng_5_, five Ng atoms progressively bind to one of the two equivalent ends of the Ng-H-Ng^+^ core, and the ensuing ring is eventually “capped” by a further Ng. A second specular capped ring of six Ng atoms is then obtained around the other Ng of the core, and the cluster eventually looks like a “roller” made up of an axel with three wheels, the two outside ones having hubcaps (Ritschel et al., 2005). On the other hand, consistent with previous results (Kojima et al., 1992; Bartl et al., 2013), recent experiments (Lundberg et al., 2020) showed that, in the mass spectra of protonated helium clusters, the only observed magic structure associated with the icosahedral motif is the (Ng-H-Ng^+^)Ng_11._ The (Ng-H-Ng^+^)Ng_5_ and (Ng-H-Ng^+^)Ng_17_ are missing, but there is evidence for peculiarly stable (Ng-H-Ng^+^)Ng_4_ and (Ng-H-Ng^+^)Ng_9_, the latter being the strongest abundance anomaly. Recent theoretical calculations (Császár et al., 2019) actually suggest the stability of the (Ng-H-Ng^+^)Ng_4_, and the (Ng-H-Ng^+^)Ng_11_, but do not provide evidence for peculiarly stable (Ng-H-Ng^+^)Ng_9_. Clearly, there is still room for further investigation.

The Ng-H distances of the Ng-H-Ng^+^ are experimentally unknown, and Table 1 quotes accurate available theoretical predictions (Kim and Lee, 1999; Tan and Kuo, 2019). Likewise, the NgH^+^, the values increase from He-H-He^+^ to Xe-H-Xe^+^, but are comparatively longer than those of the diatomic ions. In fact, in the Ng-H-Ng^+^, the proton is “shared” between two equivalent Ng atoms. In any case, the bonding analysis (Borocci et al., 2011) is again suggestive of covalent bonds.

Mixed ions Ng-H-Ng'^+^ are still elusive in the gas phase, and the only experimentally-detected species is the Xe-H-Kr^+^ identified in solid hydrogen by a ν_3_ absorption at 1,284 cm^−1^ (Tsuge et al., 2015). Compared with the ν_3_ of Kr-H-Kr^+^ and Xe-H-Xe^+^ measured in the same environment (see Table 1), this value is blue-shifted, and this is in line with previous theoretical predictions (Lundell, 1995) showing that in the Xe-H-Kr^+^, the proton is more tightly bound to the Xe atom and the ion is best described as a (Xe-H^+^–Kr) complex with a ν_3_ nearer to, but smaller than, the stretching frequency of XeH^+^ (2270 cm^−1^). Indeed, an asymmetric structure is a common feature of the mixed Ng-H-Ng'^+^ (Fridgen and Parnis, 1998a,b; Lundell et al., 1999). The (formal) H^+^ is more tightly bound to the heaviest Ng, and the resonance structure (Ng-H^+^–Ng') becomes progressively prevailing by increasing the difference between the proton affinity (PA) of Ng and Ng' (Ng > Ng'). Simplest illustrative examples are the Ne-H-He^+^, Ar-H-He^+^, and Ar-H-Ne^+^ reported by Koner et al. (2012). The Ne-H (1.108 Å) and He-H (0.959 Å) distances of Ne-H-He^+^ are shorter and longer, respectively, than the bond distances of Ne-H-Ne^+^ (1.139 Å) and He-H-He^+^ (0.926 Å), and the loss of Ne from Ne-H-He^+^ resulted more endothermic than that of He (18.2 vs. 12.0 kcal mol^−1^). In addition, the ν_3_ absorption of Ne-H-He^+^, 1,644 cm^−1^, falls in between the value predicted for He-H-He^+^ (1,717 cm^−1^) and Ne-H-Ne^+^ (1,437 cm^−1^). In the Ar-H-He^+^ and Ar-H-Ne^+^, the structural asymmetries are so pronounced to support their description as complexes of ArH^+^ weakly bound to He and Ne (by ca. 2.0 and 4.1 kcal mol^−1^, respectively).

Are there prospects to detect the Ng-H-Ng^+^ and Ng-H-Ng'^+^ in outer space (Fortenberry, 2017; Stephan and Fortenberry, 2017)? Assuming that the extreme conditions of some dense regions of the interstellar medium and planetary atmospheres may be sufficient to produce not only ArH^+^ but also NeH^+^, Fortenberry (2017) suggested the conceivable formation of Ar-H-Ar^+^, Ne-H-Ne^+^, and Ar-H-Ne^+^ from the exothermic reaction of NgH^+^ with the H${}_{3}^{+}$(Ng) (Ng = Ne, Ar) (*vide infra*) or with Ne or Ar atoms absorbed on polycyclic aromatic hydrocarbons (PAHs) (Rodríguez-Cantano et al., 2017). While the former process is probably hampered by the high barriers arising from the positive charges of the colliding partners, the PAHs route seems more plausible, especially in view of the predicted exothermic character of model reactions between NeH^+^ and C_10_H_8_-Ng or C_32_H_14_-Ng (Ng = Ne, Ar) (Fortenberry, 2017). Other suggestions for conceivable formation routes come from studies performed in cold matrices (Feldman et al., 2016; Saenko and Feldman, 2016), showing that ionized molecules such as H_2_O and CH_3_OH (quite abundant in the outer space) might protonate Ng atoms so to eventually form the Ng-H-Ng^+^.

## (H${}_{2}^{+}$)(Ng)*_*n*_* (*n* ≥ 1)

The NgH${}_{2}^{+}$, particularly the lightest HeH${}_{2}^{+}$, NeH${}_{2}^{+}$, and ArH${}_{2}^{+}$, have received sustained experimental and theoretical interest, also in connection with their role in reactions (3) and (4). Illustrative in this regard are, for example, the extensive studies on the reaction between H${}_{2}^{+}$ and He performed so far by Herman, Zülicke, and their coworkers (Schneider et al., 1976; Havemann et al., 1978, and references cited therein) with crossed-beam experiments and quasi-classical trajectory calculations. The NgH${}_{2}^{+}$ possess linear Ng-H-H^+^ connectivities, and their potential wells (see Table 1) are deep enough (Theis et al., 2015; Koner et al., 2019) to sustain numerous vibrational and rovibrational states. The latter were accurately estimated even recently (Theis et al., 2015; Papp et al., 2017, 2018; Szidarovszky and Yamanouchi, 2017), also in the intriguing prospect of actual detection of natural NgH${}_{2}^{+}$. Some general warnings in this regard come, however, from the information already available, in particular on the gas-phase ion chemistry of Ar/H_2_ mixtures (Bedford and Smith, 1990; Hvistendahl et al., 1990; Koyanagi et al., 2000). The ArH${}_{2}^{+}$ was actually detected (Bedford and Smith, 1990) from the ligand-switching between Ar${}_{2}^{+}$ and H_2_, exothermic by ca. 17 kcal mol^−1^. However, dynamics calculations on the reaction between Ar and H${}_{2}^{+}$ (Liu et al., 2011; Hu et al., 2013) point to a direct reactive mechanism, and the experiments also showed (Bedford and Smith, 1990) that, at least down to 80 K, Ar^+^ reacts with H_2_ to directly from ArH^+^, with no evidence for ArH${}_{2}^{+}$. But even assuming stabilization at the lowest temperatures, ArH${}_{2}^{+}$ could exothermically react with H_2_ (by ca. 8 kcal mol^−1^) to form ArH${}_{3}^{+}$. The latter is stable enough to conceivably exist at the lowest temperatures of the interstellar medium (*vide infra*).

The NgH${}_{2}^{+}$ are the simplest members of the H${}_{2}^{+}$(Ng)_*n*_ family (*n* ≥ 1). The H${}_{2}^{+}$(Ng)_2_ were, in particular, theoretically investigated by Krapp et al. (2008), who obtained evidence for linear symmetric complexes (Ng-H-H-Ng)^+^, thermochemically bound with respect to H${}_{2}^{+}$ and two Ng (see Table 1). However, this stability must contend with the rearrangement into Ng—NgH${}_{2}^{+}$ that resulted exothermic for Ng = Xe, and very fast for Ng = He and Ne. Therefore, the experiments were oriented to search the argon and krypton congeners; however, no Ar_2_H${}_{2}^{+}$ or Kr_2_H${}_{2}^{+}$ were detected from ionized Ng/H_2_ mixtures (Krapp et al., 2008). Somewhat unexpectedly, the lightest H${}_{2}^{+}$(He)_2_ was instead observed by electron ionization of H_2_-doped helium droplets soon afterward (Jaksch et al., 2009). Besides the expected dissociation into HeH${}_{2}^{+}$ and He, featuring a low kinetic energy release (KER) of 15 ± 4 meV, the mass-analyzed kinetic energy experiments unraveled a not-surmised dissociation into HeH^+^ and HeH (or He + H), occurring with a higher probability, and a KER four times larger than that of the loss of He. This behavior was ascribed to a metastable, electronically excited H${}_{2}^{+}$(He)_2_, whose excess energy arising from the ionization event allows the rupture of the stronger H-H^+^ bond, the weaker He-H^+^ remaining intact. Larger H${}_{2}^{+}$(He)_*n*_ (*n* ≤ 30) were subsequently detected from ionized helium droplets doped with H_2_ (Bartl et al., 2013), but their structure is only little explored.

## (H${}_{3}^{+}$)(Ng)*_*n*_* (*n* ≥ 1)

The H${}_{3}^{+}$(Ng)_*n*_ generally consist of a H${}_{3}^{+}$ covalent ionic core weakly bound to one or more Ng atoms. According to the calculations (Beyer et al., 1999; Kaczorowska et al., 2000), the first three Ng add to the vertices of the equilateral H${}_{3}^{+}$, and the fourth and the fifth ones complete the bi-pyramidal structure shown in Figure 1. The vertex-coordination of H${}_{3}^{+}$(Ar) is also supported by spectroscopic measurements (Bogey et al., 1987, 1988; Bailleux et al., 1998; McCarthy and Thaddeus, 2010). As shown in Table 1, the addition of one Ng to H${}_{3}^{+}$ is, invariably, exothermic, the stability increasing when going from H${}_{3}^{+}$(He) to H${}_{3}^{+}$(Xe) (Savić et al., 2015; Pauzat et al., 2016). Interestingly, as shown by the calculations (Beyer et al., 1999; Mousis et al., 2008), this trend mirrors a structure of the H${}_{3}^{+}$(Ng) that gradually changes from nearly pure H${}_{3}^{+}$(He) and H${}_{3}^{+}$(Ne) to a description close to XeH^+^(H_2_). This parallels a PA of H_2_, 100.9 kcal mol^−1^, that is lower than the PA of Xe (119.4 kcal mol^−1^). In essence, the periodic increase of the stabilities of the H${}_{3}^{+}$(Ng) reflects not only the increase of the polarizability of Ng (with consequent increase of the ensuing charge transfer to H${}_{3}^{+}$), but also the onset of covalency in the Kr-H and especially the Xe-H bonds. The calculations indicate also (Pauzat and Ellinger, 2005; Pauzat et al., 2009; Chakraborty et al., 2010) that, when going to the larger H${}_{3}^{+}$(Ng)_*n*_ (*n* ≥ 2), the energy change of the reaction H${}_{3}^{+}$(Ng)_*n*_ → H${}_{3}^{+}$(Ng)_n−1_ + Ng tends to decrease by increasing *n*, appreciable jumps in particular being predicted between *n* = 1 and *n* = 2, and between *n* = 3 and *n* = 4. These trends are well-consistent with the experimental binding energies of H${}_{3}^{+}$(Ar)_*n*_ (*n* = 1-7), measured (Hiraoka and Mori, 1989) as 6.7 (*n* = 1), 4.6 (*n* = 2), 4.3 (*n* = 3), 2.5 (*n* = 4), 2.3 (*n* = 5), 2.2 (*n* = 6), and 1.6 kcal mol^−1^ (*n* = 7), and clearly mirror the growing mode of the cluster.

The vibrational and rotational patterns of the H${}_{3}^{+}$(Ar) and H${}_{3}^{+}$(Ne) were more recently refined by theoretical calculations (Theis and Fortenberry, 2015), performed also to best guide the conceivable detection of these ions in the outer space. The natural role of the H${}_{3}^{+}$(Ng)_*n*_ was first suggested by Pauzat, Ellinger, and their coworkers, who proposed that the deficit of noble gases observed in planetary objects could be due to the sequestration by H${}_{3}^{+}$ during the early stages of the solar nebula (Pauzat and Ellinger, 2005, 2007; Mousis et al., 2008; Pauzat et al., 2009, 2013). According to their recent quantum-dynamics calculations (Pauzat et al., 2016), especially for the heaviest Kr and Xe, the rate constants of the radiative association are indeed large enough to support the sequestration by H${}_{3}^{+}$. The helium complexes H${}_{3}^{+}$(He)_*n*_ are also of potential astrochemical interest. Theoretical calculations (Chakraborty et al., 2010) confirm the planar structure of the H${}_{3}^{+}$(He)_3_, the three He bound to the vertices of H${}_{3}^{+}$ featuring nearly constant complexation energies of ca. 1 kcal mol^−1^. On the experimental side, Gerlich and his coworkers (Savić et al., 2015) recently produced H${}_{3}^{+}$(He)_*n*_ (*n* up to 9) in an ion trap cooled down to 3.7 K. Particularly for the simplest H${}_{3}^{+}$(He), laser-induced dissociation experiments unraveled almost 100 lines between 2,700 and 2,765 cm^−1^, whose detailed assignment was, however, hampered by the lack of a sufficiently accurate potential energy surface. Species with higher *n* are also detected in different ion sources (Kojima et al., 1992; Bartl et al., 2013), the experiments suggesting, in particular, the peculiar stability of the H${}_{3}^{+}$(He)_12_, and the “magic” role of *n* = 9 and 10. Further structural assay awaits more detailed theoretical investigations.

Finally, it is worth mentioning a group of complexes of the NgH^+^ with simple molecules such as N_2_, CO, SiO, CS, BF, N_2_, and H_2_O investigated by Ghanty and his coworkers (Jayasekharan and Ghanty, 2008, 2012; Ghosh et al., 2013, 2014; Sirohiwal et al., 2013; Sekhar et al., 2015). The HNgNH${}_{3}^{+}$ were as well-reported (Gao and Sheng, 2015). The most stable complex between NgH^+^ and a ligand *L* is the Ng-H-*L*^+^, the proton being typically more tightly bound to *L* (the PA of most molecules is, in fact, higher than that of the Ng atoms). In any case, the thermochemically less stable H-Ng-*L*^+^, best described by the limiting resonance structure (HNg^+^)*L*, is bound with respect to NgH^+^ + *L*, and kinetically protected toward the fast decomposition into Ng + *L*H^+^. Whether these conditions are sufficient for their formation in the outer space is another intriguing question related to the fascinating gas-phase chemistry of cationic noble-gas hydrides.

## Author Contributions

The author made a direct intellectual contribution to the work.

## Conflict of Interest

The author declares that the research was conducted in the absence of any commercial or financial relationships that could be construed as a potential conflict of interest.
